# Preparation and Immune Effect of HEV ORF2 P206@PLGA Nanoparticles

**DOI:** 10.3390/nano12040595

**Published:** 2022-02-10

**Authors:** Yifei Yang, Zhenning Sun, Huopeng Li, Jijing Tian, Mingyong Chen, Tianlong Liu

**Affiliations:** College of Veterinary Medicine, China Agricultural University, No. 2 West Road Yuanmingyuan, Beijing 100193, China; baiyi@cau.edu.cn (Y.Y.); zhenningsun@126.com (Z.S.); li1255880852@163.com (H.L.); jjtian@cau.edu.cn (J.T.)

**Keywords:** hepatitis E virus, vaccine immunity, nanoparticles, PLGA, P206

## Abstract

The hepatitis E virus (HEV) is an important pathogen that threatens global public health. One-third of the world’s population lives in the epidemic area of HEV, causing 20 million infections and 70,000 deaths annually. In China, HEV transmission has changed from human-to-human transmission of HEV1 to zoonotic transmission of HEV4, causing hepatitis outbreaks throughout the country. Protecting vulnerable groups, such as practitioners related to animal husbandry and downstream consumers who are immune deficient or pregnant, from HEV infections is an urgent task. At present, the commercial human vaccine, Hecolin^®^ (HEV 239 vaccine), is licensed for use only in China. HEV 239 vaccine is a human vaccine developed for HEV1. Although it has a cross-protective effect on HEV4, the level of immune protection is still different. To address the transformation of domestic HEV transmission modes, there is an urgent need to develop a new vaccine against zoonotic HEV4. P206@PLGA is a vaccine candidate in which nanomaterials are used to encapsulate viral capsid proteins for the immunization of livestock animals. Our experiments show that P206@PLGA has excellent biocompatibility and safety. In addition, P206@PLGA can effectively induce animals to produce a high titer of antibodies against HEV4, and thus has the potential to become a veterinary vaccine for the prevention of HEV. This approach provides a new concept for HE prevention to reduce the transmission of HEV in farms and protect susceptible populations.

## 1. Introduction

The hepatitis E virus (HEV) is a small, single-stranded positive-sense RNA virus with a gene sequence length of approximately 7.2 kb. HEV belongs to the family *Hepeviridae*, including *Orthohepevirus* and *Piscihepevirus*. *Orthohepevirus* A has eight genotypes [1]. At present, most studies focus on types 1–4, of which types 1 and 2 only infect humans, and types 3 and 4 are zoonotic. The genome contains three independent open reading frames (ORFs): ORF1, ORF2, and ORF3 [2]. The non-structural protein encoded by ORF1 participates in the replication of HEV RNA, ORF2 encodes the structural protein of HEV and constitutes the icosahedral capsid of the virus [3], and ORF3 protein is mainly related to the release of the virus [4]. In developed countries, human HE is prominently caused by zoonotic HEV3 and HEV4 infections, and all cases infected with HEV1 have a travel history [5]. Meanwhile the epidemic subtypes of HEV in developing countries are HEV1 and HEV2. According to a survey, the epidemic mode of HEV in China has changed from the widespread epidemic of HEV1 to the low-fluidity mode of sporadic HEV4 infection [6]. Waterborne transmission is one of the most important transmission routes of HEV, and the outbreak of HEV usually occurs after natural disasters, such as floods and rainstorms [5,7]. Recently, the risk of HEV transmission has increased significantly owing to sudden rainstorm disasters in Henan Province, China. Vulnerable groups, including immunocompromised patients, pregnant women, and the elderly, are more likely to be exposed to HEV. Moreover, the positive rate of blood HEV antibody in workers employed in animal husbandry production, slaughterhouses, or forestry is higher than that of ordinary people [8,9,10]. Therefore, there is a pressing need to prevent HEV infection among susceptible people and related workers in China.

In the case of inadequate basic sanitation facilities and poor population awareness of how to prevent HEV infection, safe and effective vaccination is still the main way to control the spread of HEV. Hecolin^®^ (HEV 239 vaccine), the first commercial HEV vaccine for human use, was approved for marketing in 2012 and is currently only allowed in China [11,12]. However, HEV4, with zoonotic characteristics, has become the main epidemic strain owing to the change in the transmission mode of HEV in China. HEV4 is widespread in livestock farms and further infects related practitioners and downstream consumers. Therefore, the development of a veterinary vaccine against HEV4 has become a new approach to reduce the spread of HEV in farms and prevent HEV infection in susceptible people.

Polymer nanoparticles (PNPs) are ideal drug carriers that have emerged in recent years. PNPs can enhance the bioavailability of drugs, prolong the drug action time, and improve drug targeting by encapsulating drugs [13], where poly(lactic-co-glycolic acid) (PLGA) is one of the most promising nanomaterials [14]. In organisms, PLGA can be completely degraded into CO_2_ and H_2_O through the tricarboxylic acid cycle. PLGA has been approved by the Food and Drug Administration (FDA) and European Medicines Agency [15]. PLGA is a copolymer synthesized from glycolic acid and lactic acid monomers through a polycondensation reaction and ring-opening polymerization. PLGA can protect drugs from degradation during the delivery process and enable slow release after reaching the site of action [16]. The literature data show that vaccines prepared using PLGA as a carrier can effectively reduce adverse reactions in immunized animals; moreover, the injection dose can be reduced and the immune effect is improved [17].

**Scheme 1:**
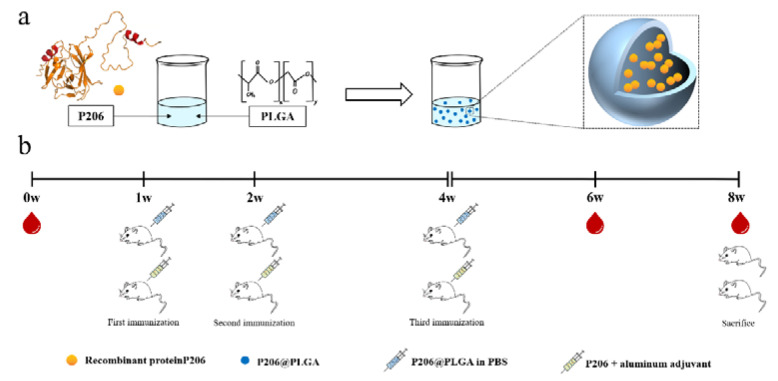
Schematic of experimental setup. (**a**) Preparation of P206@PLGA. (**b**) P206@PLGA and P206 plus aluminum adjuvants were, respectively, applied to immunize BALB/c mice, the intraocular canthal venous blood was collected at 0, 6, and 8 weeks.

In the present study, the swine-derived HEV4 HB-S4 strain is used to design an immunogenic recombinant protein, P206, which is expressed by an *E. coli* expression system. PLGA is used to wrap the HEV ORF2 recombinant protein P206 to prepare nanoparticles (P206@PLGA) with a uniform size and stable quality. Animal immunization experiments confirm that the P206@PLGA vaccine efficiently induces the production of HEV IgG antibodies in immunized animals (Scheme 1). In summary, it is confirmed that P206@PLGA has the potential to become a safe and effective animal vaccine for the prevention and treatment of HEV.

## 2. Materials and Methods

Viruses and Consumables. The virus strain was swine-derived HEV4 type HB-S3; the accession number on PubMed is KX531115.1. The pET28a (+) plasmid and expression strain, BL21, were synthesized and transformed by GenScript Corporation of the United States. The drugs used to prepare the Luria–Bertani (LB) medium were purchased from Shanghai Aladdin Biochemical Technology Co., Ltd., Shanghai, China. A His tag protein purification kit and SDS-PAGE related reagents were purchased from Shanghai Bi Yuntian Biotechnology Co., Ltd., Shanghai, China. The anti-His antibody was purchased from GenScript, New Jersey, United States. PLGA was purchased from Jinan Daigang Biological Engineering Co., Ltd., Jinan, China. Polyvinyl alcohol (PVA) was purchased from Sigma-Aldrich. Cell culture medium and fetal bovine serum were purchased from Gibco, and MTT and trypsin were purchased from Solarbio, China. The HEV IgG detection kit was purchased from Beijing Wantai Biotech.

Synthesis and Purification of P206 Protein. The LB medium was configured for the activation of strain BL21 and cultured overnight at 200 rpm and 37 °C in a shaker. The next day, the bacterial solution was removed at a ratio of 1:20, and the LB medium was preheated to 37 °C. The medium containing kanamycin sulfate was incubated at 37 °C for 30–60 min until the OD value of the bacterial solution reached 0.5–0.7. Isopropyl-β-d-thiogalactoside (IPTG) was added to a final concentration of 1 mM, and culturing was continued for 4–5 h. Thereafter, the bacterial liquid was collected, centrifuged at 4000× *g* at 4 °C for 20 min, the supernatant was discarded, and the precipitate was collected. The bacteria were resuspended with a precipitate:lysate ratio of 1:4; lysozyme (1 mg/mL) was added, and the mixture was placed in an ice-water bath to lyse for 30 min. An ultrasonic cell disruptor was used to lyse the bacteria on ice; the mixture was centrifuged at 10,000× *g* for 10 min at 4 °C, and the supernatant was collected. The nickel ion adsorption column was equilibrated and the lysate supernatant was slowly pumped into the adsorption column through a peristaltic pump. The protein components attached to the adsorption column were eluted with a gradient concentration of imidazole solution. SDS-PAGE was used to run the gel for identification.

Identification of P206 by Western Blotting. The bacterial solution was centrifuged at 4000 rpm for 10 min at 4 °C. The resulting pellet was mixed with 5× protein loading buffer, heated in boiling water for 10 min, centrifuged at 7000 rpm for 5 min, and the supernatant was collected. After SDS-PAGE, the proteins were transferred to polyvinylidene fluoride (PVDF) membranes (200 mA, 50 min) and blocked in 5% skimmed milk for 1 h. Anti-His and anti-HEV ORF2 antibodies were incubated at 4 °C overnight. The cells were washed with PBST three times the next day, incubated with HRP-labeled secondary antibodies at room temperature for 1 h, and then exposed and photographed.

Preparation and Scanning Electron Microscope Observation of P206@PLGA. The preparation method is consistent with that described in Section 3.2. The prepared P206@PLGA was stored at 4 °C for one week. An appropriate amount of P206@PLGA sample was dissolved in deionized water, ultrasonically dispersed into a suspension, and evenly dropped onto a tin foil. A magnetron sputtering machine was used for gold spraying, and a scanning electron microscope (HITACHI, Tokyo, Japan) was used to take pictures with different magnifications of the same field of view.

MTT assay of P206@PLGA. Huh7 cells (10^3^ per well) were seeded into a 96-well plate, and 200 μL of DMEM with 10% fetal bovine serum was added to each well. After the cells adhered to the wall, the medium was replaced with a different concentration of P206@PLGA medium and incubated at 37 °C under 5% CO_2_ for 24 h, after which MTT was added. After incubation for 4 h, the culture medium was discarded, the culture was terminated, 150 μL DMSO was added to each well, and the wells were shaken for 10 min in the dark. The absorbance of each well was measured using a microplate reader, and the results were recorded. The formula for calculating the cell growth inhibition rate (IR) and relative survival rate is as follows: IR = (1 − *A*_experimental group_/*A*_control group_) × 100%; relative survival rate = 100%—IR.

Evaluation of the Effectiveness of P206@PLGA. The grouping and immunization of mice were consistent with those described in Section 3.4. After collecting blood from the intraocular canthus of the mice, the sample was first placed at room temperature for 1 h, then allowed to stand at 4 °C for 1 h, and finally centrifuged at 5000 rpm for 10 min. The supernatant was collected. The HEV IgG kit was used for the detection. The secondary antibody was replaced with an HRP-labeled secondary antibody. After adding the color developing solution, the cells were incubated at 37 °C for 15 min in the dark, and the absorbance of each well was measured at 450 nm wavelength after termination. The antibody titer was determined as described above. First, the serum sample was diluted to 10^1^–10^9^, and the HEV IgG kit was used for detection. The antibody titer was calculated using *P/N* ≥ 2 as the standard.

Statistical analysis. All charts in this experiment were drawn using GraphPad Prism 8, and statistical analysis was performed using SPSS (Statistical Product and Service Solutions). All experimental data are presented as the mean ± SD. A two-way ANOVA was used to compare multiple experimental groups. The statistical significance was set at *p* < 0.05; * *p* < 0.05.

## 3. Results

### 3.1. Expression and Identification of Recombinant Protein P206

**Figure 1:**
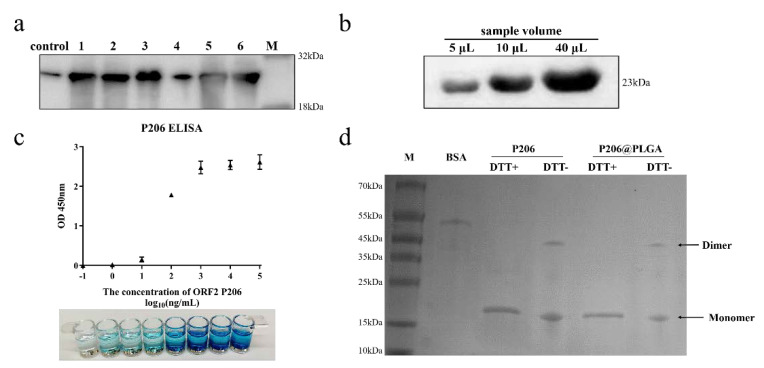
Identification of recombinant protein P206; (**a**) expression of P206 under different induced conditions (control: without IPTG induction; 1: input, 15 °C 16 h; 2: input, 37 °C 4 h; 3: inclusion body, 15 °C 16 h; 4: supernatant, 15 °C 16 h; 5: inclusion body, 37 °C 4 h; and 6: supernatant, 37 °C 4 h). (**b**) Western blot data for different P206 concentrations combined with anti-ORF2 antibody; (**c**) OD value of protein P206 at 450 nm and TMB staining; and (**d**) native PAGE data for protein P206 and PLGA@P206.

The concentration of purified P206 was 5.2 mg/mL; P206 was detected by using anti-His tag antibody. The results of Western blot (WB) analysis showed obvious protein bands at 23 kD for each sample group, indicating that the *E. coli* expression system successfully expressed P206. The expression of P206 in bacterial inclusion bodies was the highest under the induction conditions of 15 °C for 16 h (Figure 1a). To further detect the antigenicity of P206, the anti-HEV ORF2 antibody was incubated with different concentrations of P206 (5, 10, and 40 μg). The WB results show that P206 is capable of binding to the anti-HEV ORF2 antibody and has antigenicity, and the protein concentration is positively correlated with the amount of bound antibody (Figure 1b). The indirect ELISA method was used to detect the absorbance of P206 at a wavelength of 450 nm. At concentrations from 10 ng/mL to 10^2^ ng/mL, P206 showed good antigenicity (Figure 1c). Native PAGE analysis showed bands at both 23 kDa and 46 kDa, indicating that P206 could self-assemble into dimers under the condition that the quaternary structure of the protein was retained. It was preliminarily confirmed that P206 could form VLPs (Figure 1d). 

### 3.2. Preparation and Characterization of P206@PLGA

**Figure 2:**
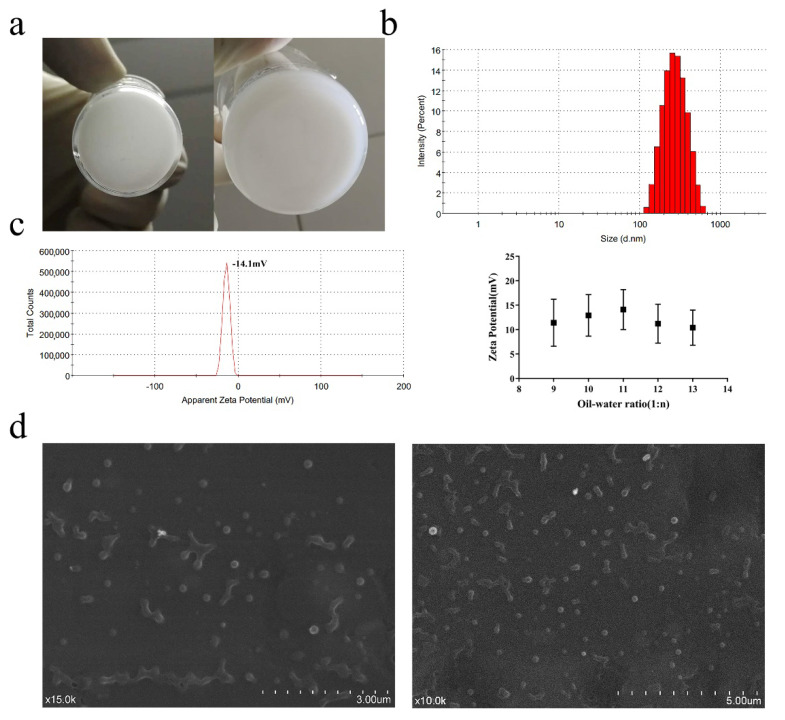
Preparation and characterization of P206@PLGA NPs. (**a**) First and multiple W/O/W emulsions of P206@PLGA NPs; (**b**) particle size and (**c**) zeta potential of synthesized P206@PLGA NPs and zeta potential at different oil–water ratios; and (**d**) SEM image of P206@PLGA NPs.

An appropriate amount P206 powder was dissolved in 3% NaCl to prepare a 1 mg/mL P206 solution as the internal water phase (W1). A 1% PVA solution was used as the outer water phase (W2), and a 0.5% PVA solution was used as the diffusion phase. PLGA (200 mg) was dissolved in different volumes of ethyl acetate (EtAc) as an organic solvent (with different water–oil ratio). After ultrasonic dispersion, PLGA was used as the oil phase (O). The appropriate amount of the internal water phase was slowly dropped into the oil phase and ultrasonicated for 8 min in an ice bath (working for 2.5 s, pause for 2.5 s) to obtain the first emulsion. The prepared first emulsion was slowly added to 10 mL of 1% PVA aqueous phase, and then ultrasonicated for 15 min in an ice bath (working for 3 s, pause for 3 s) to obtain multiple emulsions (Figure 2a). The mixture was stirred for 3–4 h to volatilize the oil phase, and washed 3 times to obtain P206@PLGA nanoparticles. The particle size of the synthesized P206@PLGA NPs was approximately 354 nm (Figure 2b). The zeta potentials of all P206@PLGA NPs at different ratios were negative. When the ratio was 1:11, the zeta potential was −14.1 ± 4.1 mV, where the absolute value was the highest among all the samples (Figure 2c). As shown in Figure 2d, the P206@PLGA NPs were spherical, uniform in size, and well dispersed. The entrapment efficiency of P206@PLGA NPs at a ratio of 1:11 was 77.46 ± 0.924%, as determined by using the bicinchoninic acid method. The treatment mentioned above did not degrade the P206 protein; the presence of dimer was still observed in the sample without DTT treatment (hole 5; Figure 1d), indicating that the integrity and effectiveness of the P206 protein were retained in the P206@PLGA NPs.

### 3.3. Safety Evaluation of P206@PLGA

**Figure 3:**
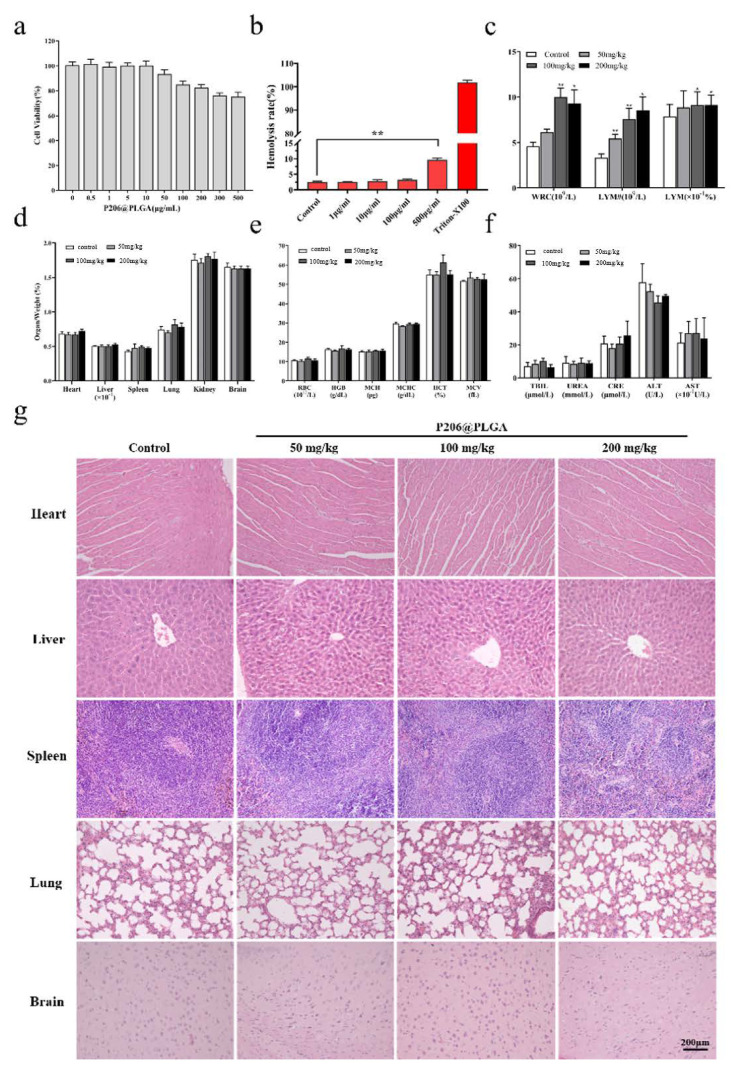
Safety evaluation of P206@PLGA. (**a**) MTT assay of P206@ PLGA in Huh7 cells; (**b**) hemolysis test using P206@ PLGA; (**c**) changes in WBC and lymphocyte, (**d**) organ/weight ratio, (**e**) RBC related indicators, and (**f**) serum biochemical indicators with different concentrations of P206@PLGA; (**g**) hematoxylin and eosin staining of major organs; hemosiderin deposition could be seen in the spleen of mice in the high-dose P206@PLGA (200 mg/kg) group. The scale bar corresponds to 200 μm.

Different concentrations of P206@PLGA (0.5–500 μg/mL) were co-cultured with Huh7 cells for 24 h, and the cytotoxicity was detected by MTT assay. P206@PLGA did not inhibit cell growth when the concentration of P206@PLGA was 0.5−10 μg/mL; the cell survival rate decreased with increasing drug concentration (Figure 3a). The cell survival rate was approximately 75% at a concentration of 500 μg/mL, and cell growth was still not significantly inhibited (the cell survival rate was less than 70%). This observation indicated that P206@PLGA exhibited excellent safety in in vitro cell experiments. The red blood cell hemolysis test showed that P206@PLGA did not exhibit hemolysis in the concentration range of 1–100 μg/mL (Figure 3b), and no red blood cell abnormalities were observed under a microscope (Figure S4). P206@PLGA groups with 3 different concentrations (50, 100, and 200 mg/kg) were administered to ICR mice via tail vein injection. No significant changes were found in indicators, such as the organ/weight ratio, serum biochemistry, and blood routine (Figure 3d–f), while the lymphocyte counts increased significantly (Figure 3c). Moreover, tissue sections of the heart, liver, spleen, lung, and brain of each group of mice showed no obvious lesions (Figure 3g).

### 3.4. Evaluation of the Effectiveness of P206 and P206@PLGA Nanospheres

**Figure 4:**
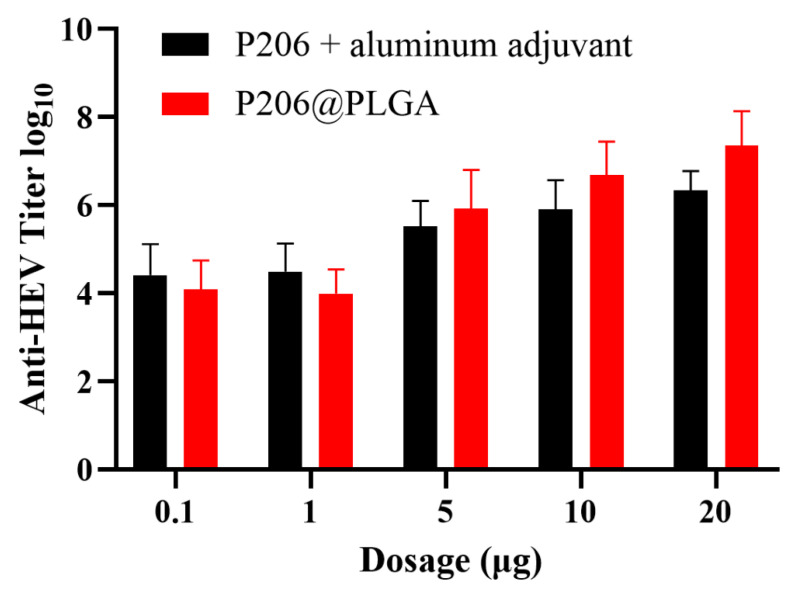
Determination of anti-HEV antibody titer in the serum of BALB/c mice eight weeks after immunization with P206 plus aluminum adjuvant and P206@PLGA. The antibody titer induced by P206@PLGA in the 20 μg group was significantly higher than that in the P206 plus aluminum adjuvant group (*p* < 0.05).

Thirty BALB/c mice were randomly divided into five groups and administered different doses of P206 and P206@PLGA. P206 and aluminum adjuvant were mixed in a 1:1 ratio and administered by multi-point injection through the back and the tail root subcutaneously. Immunizations were performed in the first, second, and fourth weeks. Blood (100 μL) from the intraocular canthus was collected before the first immunization and in the sixth week, after which the serum HEV IgG was detected. The use of P206 plus aluminum adjuvant to immunize mice could increase the positive conversion rate of HEV IgG at a lower drug dose; however, it could not stably induce the appearance of HEV IgG in 100% of the mice as the concentration of P206 increased (Table 1). In contrast, the positive rate of HEV IgG in the blood of mice could be stabilized at 100% after immunization with high doses of P206@PLGA (10 μg and 20 μg), and the resulting antibody titer was approximately 10 times that induced by protein P206 plus aluminum adjuvant (Figure 4). The weight change and the weight ratio of the organs to the weight of the mice during the immunization process were also tested, demonstrating that the P206@PLGA-treated group performed well during and after immunization (Figure S6).

## 4. Discussion

The HEV ORF2 protein is a structural protein of the virus, which has good immunogenicity and contains a neutralizing epitope of the virus. Consequently, the HEV4 HB-S4 strain was isolated and purified from the livestock farm as the research object. In addition, we selected 429–634 amino acid (aa), which is the epitope concentration region on ORF2 of the strain, as the target fragment; this fragment was named P206. The production of protective neutralizing antibodies depends on the natural epitope on the surface of virus-like particles (VLPs) and the correct antigen conformation, which also determines the effectiveness of the vaccine. Hence, in addition to preserving the antigen-neutralizing epitope of the ORF2 protein bulge domain, attempts were made to extend the C-terminal aa sequence to improve the stability of the VLPs to form a polypeptide chain with a length of approximately 200 aa during the process of protein sequence design [18]. Moreover, a His tag was added to the N-terminus of the sequence for purification and identification of recombinant proteins. Our results reveal that recombinant protein P206 is successfully expressed and purified. The expression of P206 in bacterial inclusions was the highest under continuous low-temperature induction (15 °C, 16 h). P206 can self-assemble into a dimer form, which preliminarily proves that P206 has the ability to assemble into VLPs.

To effectively immunize animals while reducing their adverse reactions, we hope to use a low-dose, sustained slow-release immunization method to alleviate the side effects of the commonly used three-injection aluminum adjuvant vaccine. For this purpose, degradable high-molecular polymers are used as drug carriers to improve the sustained release and to enhance the stimulating effects of VLPs on the immune system. PLGA has become one of the most popular and widely used carrier materials for preparing polymer nanoparticles because of its good biocompatibility, high biosafety, low cost, and easy availability. PLGA NPs exhibit good biosafety in a variety of cell lines, and there is no obvious toxic effect in rats after oral administration [19,20]. However, considering that PLGA must be used to wrap the recombinant protein P206 in the drug delivery system, the protein polypeptide may be denatured and become toxic during this process. PLGA itself may also be biologically toxic based on the preparation process. Therefore, the safety evaluation of P206@PLGA is necessary. The above results demonstrate that P206@PLGA exhibits good biosafety, both in vivo and in vitro, which is the basis and premise for the application of P206@PLGA in clinical practice.

After testing the antigenicity of P206 protein and the safety of the P206@PLGA nanoparticles, the immune effects of the P206 protein and P206@PLGA nanospheres were tested through ELISA to explore whether P206@PLGA is a prospective candidate as an HE vaccine. Generally, adjuvants are added to traditional vaccines to stimulate the body’s innate immune response [21]. With the popularity of subunit vaccines and nucleic acid vector vaccines, the use of adjuvants is expected to further increase to compensate for the lack of immunogenicity of these vaccines [22]. In this process, the side effects and safety problems associated with vaccine become more pronounced as the dosage increases, although aluminum adjuvants and other vaccine adjuvants have good safety and have been approved for use by the FDA and EMA. In addition, this type of vaccine is often not as effective as expected in inducing cellular immunity and cytotoxic T lymphocyte (CTL) responses [23,24]. Therefore, we believe that protein P206 as a VLP has better immunogenicity on one hand, and on the other hand, it can stimulate the body to produce a more comprehensive immune response. At the same time, the PLGA nanospheres encapsulating the protein P206 can be continuously lysed in the body, slowly releasing the antigen to stimulate the body to produce a sustained immune response, where related studies have shown that this stimulating effect can last for approximately four weeks [25]. To some extent, PLGA replaces the aluminum adjuvant and improves the safety and immune effects of the vaccine. The present experimental data also indicate that compared with the protein P206 plus aluminum adjuvant immunization method, the immune effect of P206@PLGA is more stable, and the antibody titer produced is also higher.

## 5. Conclusions

In the present experiment, the HEV type 4 HB-S3 strain was used as the research object, and the target protein sequence was designed by cutting its ORF2 sequence to retain the main epitope region. The immunogenic recombinant protein P206 was successfully synthesized using an *E. coli* expression system. To improve the safety and effectiveness of the vaccine, PLGA was applied as a nanocarrier to wrap P206 to prepare nanospheres of P206@PLGA with a particle size of approximately 354 nm and a negatively charged surface. The data demonstrate that P206@PLGA exhibits good biosafety both in vivo and in vitro. Compared with the traditional vaccine comprising P206 plus an aluminum adjuvant, the immune effect of P206@PLGA is more stable and the induced antibody titer is also higher. Therefore, we believe that P206@PLGA is a prospective vaccine candidate for preventing HEV infection in livestock farms in China.

## Figures and Tables

**Table 1:** Positive conversion rate of HEV IgG in the serum of mice after immunization with P206 plus aluminum adjuvant and P206@PLGA, respectively.

Dose (μg)	P206 Plus Aluminum Adjuvant	P206@PLGA
	Positive Seroconversion/Number of Mice Vaccinated	
0.1	3/6 (50%)	1/6 (17%)
1.0	3/6 (50%)	3/6 (50%)
5.0	6/6 (100%)	4/6 (67%)
10	5/6 (83%)	6/6 (100%)
20	6/6 (100%)	6/6 (100%)

## Data Availability

Not applicable.

## Supplementary Materials

The following supporting information can be downloaded at: https://www.mdpi.com/article/10.3390/nano12040595/s1, Figure S1. Sequence of Plasmid pET28a (+)—P206; Figure S2. Expression of protein P206 under different induction conditions; Figure S3. RBC hemolysis test of different P206@PLGA concentrations; Figure S4. Microscopic morphology of red blood cells (RBCs) under different concentrations of P206@PLGA; Figure S5. Changes of BALB/c mice weight from 1 to 8 weeks after P206@PLGA immunization; and Figure S6. Organ/weight ratio of P206@PLGA immunized BALB/c mice.
