# Supplementary material for: Preparation and Immune Effect of HEV ORF2 P206@PLGA Nanoparticles
Source: Nanomaterials (Basel). 2022 Feb 10;12(4):595. doi: 10.3390/nano12040595 (PMC8878542; doi:10.3390/nano12040595)

Electronic Supplementary Information

Preparation and Immune Effect of HEV ORF2 P206@PLGA Na-noparticles

Yifei Yang, † Zhenning Sun, † Huopeng Li, Jijing Tian, Mingyong Chen, * Tianlong Liu*

[1]College of Veterinary Medicine, China Agricultural University, No.2 West Road Yuanmingyuan, Beijing 100193, P.R. China; liutianlong@cau.edu.cn
† These authors contributed equally.

*Correspondence: Mingyong Chen
College of Veterinary Medicine, China Agricultural University, No.2 West Road Yuanmingyuan, Beijing 100193, P.R. China
Email: chenmingyong@cau.edu.cn.

Figure S1 **Sequence of Plasmid pET28a (+) - P206.** ATG is the start codon and TGA is the stop codon. The front segment is the NcoI restriction site and the back segment is the HindIII restriction site.

```
1     CCATGCATCATCATCACCACCACGTAGAAAATGCTCAACAAGAT
        M  H  H  H  H  H  H  V  E  N  A  Q  Q  D
45    AAAGGAATTGCGATTCCGCACGACATTGACCTGGGCGAAAGCCG
      K  G  I  A  I  P  H  D  I  D  L  G  E  S  R
89    TGTGGTTATCCAGGACTATGATAACCAGCACGAGCAAGATCGTC
       V  V  I  Q  D  Y  D  N  Q  H  E  Q  D  R  P
133   CGACCCCGAGCCCGGCGCCGAGCCGTCCGTTCAGCGTGCTGCGT
        T  P  S  P  A  P  S  R  P  F  S  V  L  R
177   GCGAACGACGTTCTGTGGCTGAGCCTGACCGCGGCGGAATATGA
      A  N  D  V  L  W  L  S  L  T  A  A  E  Y  D
221   TCAGACCACCTACGGTAGCAGCACCAACCCGATGTACGTGAGCG
       Q  T  T  Y  G  S  S  T  N  P  M  Y  V  S  D
265   ATACCGTTACCTTTGTGAACGTTGCGACCGGTGCGCAAGGCGTG
        T  V  T  F  V  N  V  A  T  G  A  Q  G  V
309   AGCCGTAGCCTGGACTGGAGCAAAGTTACCCTGGATGGTCGTCC
      S  R  S  L  D  W  S  K  V  T  L  D  G  R  P
353   GCTGACCACCATTCAGCAATATAGCAAGACCTTCTACGTGCTGC
       L  T  T  I  Q  Q  Y  S  K  T  F  Y  V  L  P
397   CGCTGCGTGGCAAACTGAGCTTTTGGGAGGCGGGCACCACCAAG
        L  R  G  K  L  S  F  W  E  A  G  T  T  K
441   GCGGGCTACCCGTATAACTACAACACCACCGCGAGCGACCAAAT
      A  G  Y  P  Y  N  Y  N  T  T  A  S  D  Q  I
485   CCTGATTGAAAACGCGGCGGGTCACCGTGTGTGCATCAGCACCT
       L  I  E  N  A  A  G  H  R  V  C  I  S  T  Y
529   ATACCACCAACCTGGGTAGCGGTCCGGTTAGCATTAGCGCGGTG
        T  T  N  L  G  S  G  P  V  S  I  S  A  V
573   GGCGTTCTGGCGCCGCATACCGCGCTGGCGGTTCTGGAGGACAC
      G  V  L  A  P  H  T  A  L  A  V  L  E  D  T
617   CGTTGACTACCCGGCGCGTGCGTAATGAAAGCTT
       V  D  Y  P  A  R  A  .  .  K  L
```

Figure S2. Expression of protein P206 under different induction conditions.

1: 1 μg BSA; 2: 2 μg BSA; 3: bacterial lysate without IPTG induction;

4~5: inclusion body; 4: IPTG induction, 15 ℃ 16 h; 5: IPTG induction, 37 ℃ 4 h;

6~8: supernatant; 6: without IPTG; 7: IPTG induction, 15 ℃ 16 h; 8: IPTG induction, 37 ℃ 4 h;

9~11: bacteria precipitation; 9: without IPTG induction; 10: IPTG induction, 15 ℃ 16 h; 11: IPTG induction, 37 ℃ 4 h.

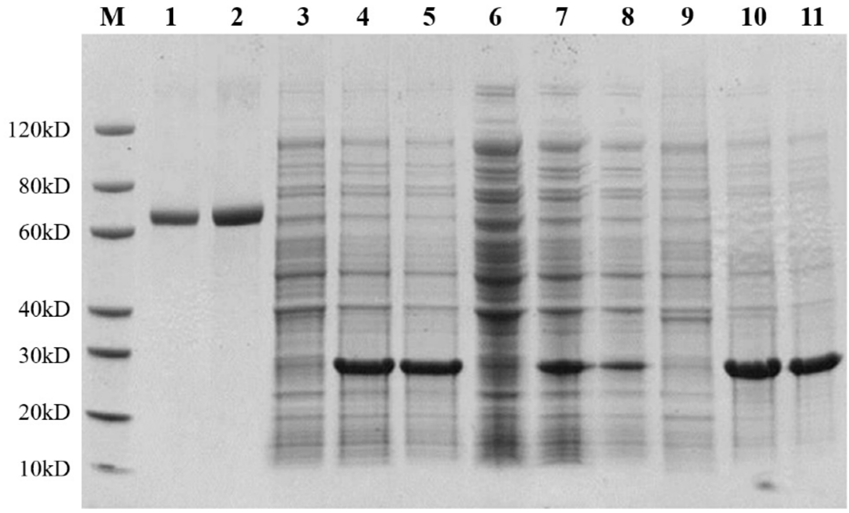

Figure S3. RBC hemolysis test of different P206@PLGA concentrations.

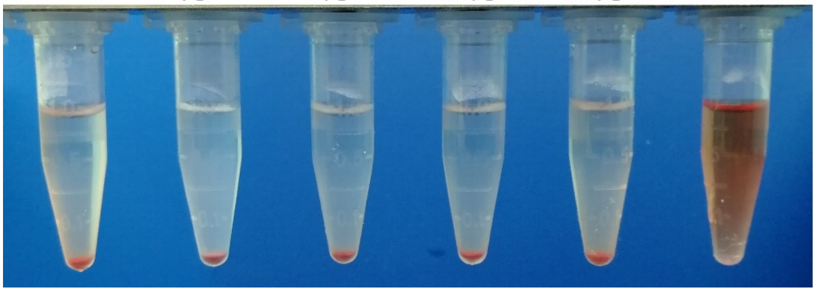

Figure S4. Microscopic morphology of red blood cells (RBC) under different concentrations of P206@PLGA. The RBC morphology under the action of different concentrations of P206@PLGA did not change significantly under the microscope. The scale bar corresponds to 50 μm.

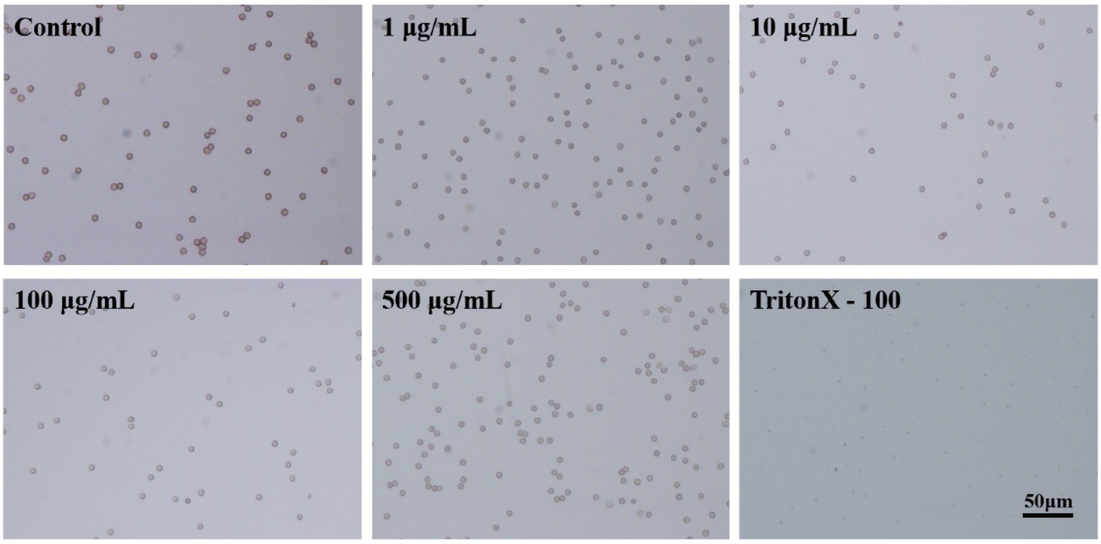

Figure S5. Changes of BALB/c mice weight from 1 to 8 weeks after P206@PLGA immunization. The body weight of the BALB/c mice gradually increased and there was no significant difference in the growth trend between the groups.

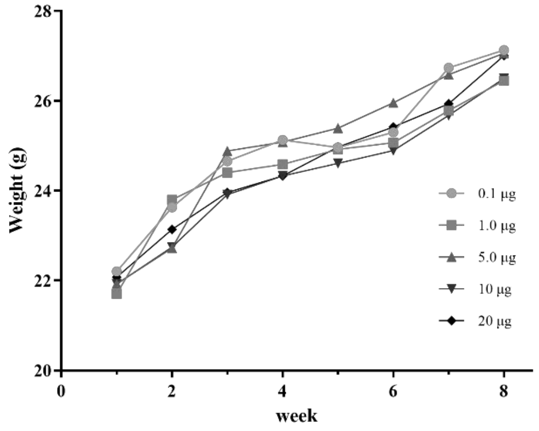

Figure S6. Organ/weight ratio of P206@PLGA immunized BALB/c mice. There was no significant difference in the ratio of each organ to body weight except for the heart weight ratio of 5, 10, 20 μg P206@PLGA group; the body weight of mice in the 1 and 5 μg P206@PLGA treatment groups decreased significantly ($P < 0.05$). (Organ/weight ratio = organ/weight × 100%)

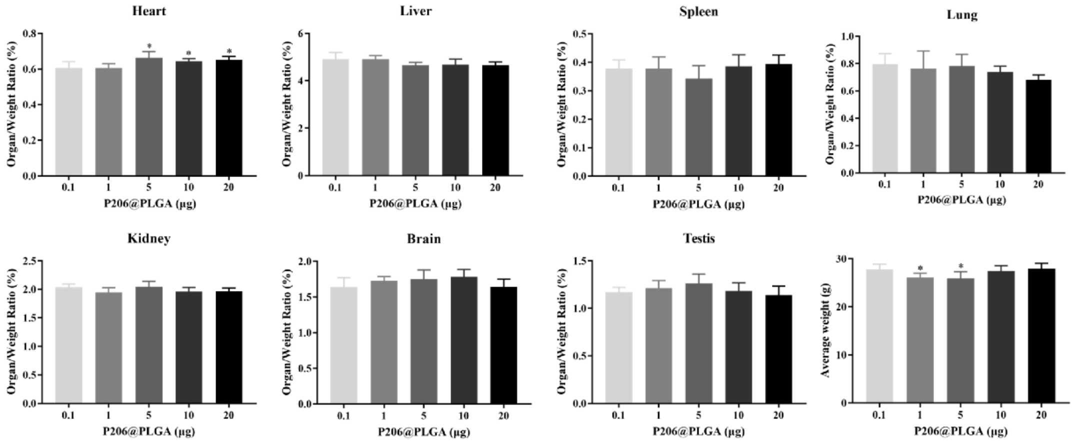

Supplement: Supplementary file 1 [file nanomaterials-12-00595-s001.zip › nanomaterials-1540037-supplementary.pdf]
